# Falls, healthcare resources and costs in older adults with insomnia treated with zolpidem, trazodone, or benzodiazepines

**DOI:** 10.1186/s12877-022-03165-6

**Published:** 2022-06-04

**Authors:** Diana T. Amari, Timothy Juday, Feride H. Frech, Weiying Wang, Zheng Wu, Norman Atkins, Emerson M. Wickwire

**Affiliations:** 1 Genesis Research, 111 River Street, Ste 1120, Hoboken, NJ 07030 USA; 2 Eisai, Inc., 200 Metro Blvd., Nutley, NJ 07110 USA; 3 University of Maryland Sleep Disorders Center, 100 N. Greene St, 2nd Floor, Baltimore, MD 21201 USA

**Keywords:** Falls, Insomnia, Utilization, Cost, Older adults, Medicare

## Abstract

**Background:**

Falls are the leading cause of injury-related death among older Americans. While some research has found that insomnia heightens falls, health care resource utilization (HCRU) and costs, the impact of insomnia treatments on fall risk, mortality, HCRU and costs in the elderly population, which could be of substantial interest to payers, has not been fully elucidated. This study evaluated the risk of falls and related consequences among adults ≥ 65 years of age treated with common prescription medications for insomnia compared with non-sleep disordered controls.

**Methods:**

This was a retrospective cohort analysis of deidentified Medicare claims from January 2011 through December 2017. Medicare beneficiaries treated for insomnia receiving zolpidem extended-release, zolpidem immediate-release, trazodone, or benzodiazepines were matched with non-sleep disordered controls. The main outcomes were falls, mortality, healthcare resource utilization (HCRU), and medical costs during the 12 months following the earliest fill date for the insomnia medication of interest. Generalized linear models controlled for several key covariates, including age, race, sex, geographic region and Charlson Comorbidity Index score.

**Results:**

The study included 1,699,913 Medicare beneficiaries (59.9% female, mean age 75 years). Relative to controls, adjusted analyses showed that beneficiaries receiving insomnia medication experienced over twice as many falls (odds ratio [OR] = 2.34, 95% CI: 2.31–2.36). In adjusted analyses, patients receiving benzodiazepines or trazodone had the greatest risk. Crude all-cause mortality rates were 15-times as high for the insomnia-treated as controls. Compared with controls, beneficiaries receiving insomnia treatment demonstrated higher estimated adjusted mean number of inpatient, outpatient, and emergency department visits and longer length of inpatient stay. All-cause total adjusted mean costs were higher among insomnia treated patients ($967 vs $454).

**Conclusions:**

Individuals receiving insomnia treatment had an increased risk of falls and mortality and higher HCRU and costs compared with matched beneficiaries without sleep disorders. Trazodone and benzodiazepines were associated with the greatest risk of falls. This analysis suggests that significant risks are associated with common, older generation insomnia medication treatments in the elderly. Nonetheless, these results should be interpreted with caution as the use of these medications may be indicative of underlying morbidity with potential for residual confounding.

**Supplementary Information:**

The online version contains supplementary material available at 10.1186/s12877-022-03165-6.

## Background

Falls are the leading cause of injury-related deaths among older adults (ie, age ≥ 65 years) [1] and incur a substantial economic burden due to costs associated with subsequent fractures, hospital visits, and long-term care [2]. In particular, medication-related falls or fractures are recognized as an important consideration for clinicians when initiating new pharmacotherapy in older adult patients. Clinical experts have published guidelines, such as the American Geriatrics Society (AGS) Beers Criteria [3], to help inform treatment decisions. However, details on guideline use when treating certain conditions are scant.

Insomnia, a common sleep–wake disorder characterized by difficulty initiating or maintaining sleep or both, is more prevalent among older adults [4, 5] and older adults are more likely to be prescribed medication for insomnia treatment [6]. Medication classes commonly used to treat insomnia – including benzodiazepines and z-drugs (i.e. non-benzodiazepine sedative hypnotics such as zolpidem) – can have adverse effects that may pose elevated risk for older adults. Benzodiazepine adverse effects include cognitive and memory impairment, rebound insomnia upon cessation, and increased risk of motor vehicle accidents (MVAs), falls, dependence, and addiction [7, 8]. Z-drugs are associated with next day cognitive, memory, psychomotor, and balance impairments and risk of dependence and addiction [7, 8]. Thus, these medications may be inappropriate for use in older adults, particularly those with a history of falls or fractures [9], due to unfavorable risk–benefit ratios. Clinicians often prescribe off-label the heterocyclic trazodone for insomnia management. Trazodone may be perceived as a safer alternative in older adults due to the lack of anti-cholinergic activity and cardiotoxicity, although limited published data are available to support efficacy or safety in this population [10]. The most common adverse effects associated with trazodone use include daytime sleepiness and orthostatic hypotension [11], which may pose a risk of falls in an older population, particularly upon awakening from sleep [12]. National data suggests that zolpidem and low-dose trazodone are the most frequently prescribed medications used for insomnia management in the US, followed by benzodiazepines; use of low-dose trazodone for insomnia has increased in recent years while zolpidem use has decreased [6, 13].

Given the aging US populace and the increasing prevalence of insomnia among older adults, insomnia-related falls represent a major public health concern [14–18]. However, no recent studies have assessed healthcare costs or outcomes associated with frequently prescribed insomnia medications among older adults.

To address this known gap in the literature, the objective of this study was to evaluate the risk of falls among older adults (age ≥ 65 years) prescribed medications commonly used to treat insomnia. Additional study objectives were to describe mortality, all-cause health care resource utilization (HCRU), and costs among older adults prescribed these medications for insomnia treatment.

## Methods

### Data source

This was a retrospective cohort study using the 100% Medicare administrative claims database (ie, research identifiable files, RIFs) from January 2011 through December 2017. The Medicare database provides data in 3 separate file types: 1) data that can allow patient identification; 2) dataset files containing patient-level data but no identifying information; 3) data that has been aggregated and contains no patient- or provider-level information. Care settings included within the Medicare database comprise outpatient, inpatient, skilled nursing facility, hospice, home health agency, prescription drugs and more. Of note, claims data generally provide information on the provision of a service (e.g., laboratory test) rather than the outcome (e.g., laboratory result) [19].

In this study, patients receiving insomnia treatment were identified from 1 January 2012 through 31 December 2016. The index date was defined as the earliest fill date for an insomnia medication of interest (benzodiazepines, trazodone, zolpidem ER (extended release), and zolpidem IR (immediate release)). Based on available market research, the most commonly used prescription insomnia medications were selected for analysis. Other prescription insomnia medications were not included, largely due to low sample size issues stemming from limited time on the market and/or low market share. In addition, a matched control cohort of non-sleep disordered adults were identified from January 2012 through December 2016. Patients in the control group were matched 1-to-1 with individuals in the insomnia treated group based on birth year and sex. For the matched control group, the index date was defined as the index date for the matched insomnia patient. 

This study was performed in accordance with the Helsinki Declaration of 1964, and its amendments. Analysis of commercially available de-identified secondary data sources is considered exempt from the requirements for “human subjects research” in the US. This study did not involve collection, use or transmittal of individually identifiable data and only used de-identified data therefore Institutional Review Board (IRB) approval was not required.

### Study population

All patients were aged ≥ 65 years at index date and were enrolled in health plans with full medical and drug coverage continuously for at least 12 months before and after the index date. The index date was the earliest prescription fill date for an insomnia medication of interest. To ensure that all patients included in the study had insomnia and were receiving prescription medication treatment for their condition, the primary inclusion was 1) ≥ 1 FDA-approved insomnia medication of interest or 2) trazodone ≤ 100 mg or ≥ 1 off-label insomnia treatment coupled with ≥ 1 physician-assigned ICD-9/ICD-10 insomnia diagnosis code within 12 months pre-index date. In addition, to ensure treatment for chronic insomnia (e.g., as opposed to a short-term hypnotic prescription for long-haul air travel), beneficiaries with claims for a single insomnia treatment were required to have received ≥ 5 days’ supply based on expert opinion. Exclusion criteria included 1) missing birth year or sex; 2) claim for an insomnia medication of interest within the 12-month pre-index period to remove prior treated insomnia patients; 3) patients receiving benzodiazepines with a concurrent diagnosis of anxiety; 4) a motor vehicle crash (MVC) concurrent to a fall event if documented within 7 days of the fall event. In such instances, motor vehicle insurance may have covered a substantial portion of healthcare costs, which could bias results. No data were available for non-prescription treatments in this dataset, including over-the-counter treatments or cognitive behavioral therapy for insomnia, and therefore these treatment options were not further considered.

Inclusion in the matched control cohort required no evidence of sleep-related disorders including insomnia, insomnia diagnosis or sleep-related disorders in Medicare claims during the observational period. Specific sleep-related disorders/medications/procedures excluded patients from the matched control cohort: (1) insomnia, hypersomnia, sleep-related breathing disorders, circadian rhythm sleep disorders, parasomnia, sleep-related movement disorders and drug-induced sleep disorders; (2) sleep-related medications, which are insomnia medications of interest in this study; and (3) sleep related procedures (ie, sleep study procedures, sleep services, home sleep apnea testing, and sleep-related durable medical equipment). 

### Outcomes

“Fall events” were defined as receipt of ≥ 1 diagnostic code for falls, hip fractures, or traumatic brain injuries (TBIs), regardless of claim position or point of service. Multiple falls, hip fractures, or TBIs on the same day or within 7 days of each other were counted as a single fall event.

“MVA events” were defined as receipt of ≥ 1 diagnostic code for a MVA. If there were diagnosis claims for both MVA and falls on the same day, priority was given to the fall event. Multiple claims for MVA within 7 days were considered part of the same MVA event.

Overall all-cause annual crude mortality rate was defined as the ratio of patient deaths to total patients ([number of deaths/patient time*365 days]/total patients) and was calculated for all patients and those with and without a fall event.

HCRU included inpatient, ED (emergency department), and outpatient visits. Inpatient visits were based on inpatient admission records. ED visits were identified as outpatient visits with ED as the place of service. HCRU categories were not mutually exclusive.

Costs associated with each HCRU category were assessed overall and for patients with and without falls. (Note these costs were assessed only for those patients with at least one HCRU claim for a specific category of HCRU. For example, the total inpatient costs represented the cost associated with inpatient visits for those with at least one inpatient visit only.) Total prescription drug costs and costs for insomnia and non-insomnia medications were reported separately. All costs were estimated per patient per year (PPPY) or per patient per month (PPPM) and were adjusted to 2019 US dollars (USD) based on the medical care component of the consumer price index (CPI) [20].

### Statistical analysis

Statistical analyses were performed using SAS Enterprise Guide version 7.1 (SAS Institute, Inc., Cary, NC).

### Baseline demographics

Means, standard deviations, medians, interquartile ranges, and minimum and maximum values are reported for continuous variables. Frequencies and percentages are reported for categorical outcomes.

### Falls

Fall events were described for the treated insomnia cohort by drug, or by drug class for benzodiazepines, and for the matched control cohort within 12 months after the index date. Time to first event was also calculated as days from index date to the first fall event. Generalized linear models with Poisson distribution while adjusting for covariates (see list below) were used to estimate adjusted incidence rates, incidence rate ratios (IRR), and 95% confidence intervals (CI) between the treated and the matched cohorts. Odds ratios with 95% CI, determined using logistic regression, were used to compare the risk of fall events between the treated and matched control cohorts. The hazard ratio for time to first event was estimated using a Cox proportional hazard model with robust variance estimation while adjusting for confounding variables. The 95% CI was also reported.

### Mortality

Impact of treated insomnia on mortality was evaluated by comparing unadjusted proportions of patients with mortality outcomes (treated insomnia vs non-sleep disordered control) using chi-square tests.

### HCRU and costs

Impact of treated insomnia on HCRU and cost was evaluated by comparing beneficiaries with treated insomnia and non-sleep disordered controls using 95% CI, without adjusting for multiplicity. Statistical significance rates (*p*-values) were also reported.

### Co-variates

Co-variates included geographic region (Northeast, North Central, South, and West); medical and psychiatric comorbidity burden (baseline Charlson Comorbidity Index [CCI] score: 1, 2, 3 +) [21]; and race (Non-Hispanic White, Black [Or African American], Hispanic, Asian/Pacific Islander, American Indian/Alaska Native, and Other). Consistent with published literature, variables identified as potential confounders affecting health outcomes were derived from claims data and, all these variables were included in each adjusted model as covariates.

## Results

### Patient demographics and characteristics

After matching, 1,699,913 patients were included in each cohort (Supplementary Fig. 1). Among the insomnia treated cohort, the proportion of patients receiving each index treatment was zolpidem IR 36.2%, trazodone 34.2%, benzodiazepines 29.1%, and zolpidem ER 0.5%.

After matching, the insomnia treated and matched control cohorts had the same sex distribution and age at index (Table 1). Relative to non-sleep disordered controls, patients with treated insomnia had a substantially higher comorbidity burden (mean CCI (1.7 [SD 2.5]) vs (0.7 [SD 1.5]). In addition, patients with treated insomnia demonstrated a slightly shorter mean duration of post-index Medicare enrollment (41 months [SD 17.3] vs 44 months [SD 17.0]). No other differences in demographic or clinical characteristics were observed (Table 1).

**Table 1:** Patient demographics and clinical characteristics

Category	Insomnia Treated Patients		Matched Control Cohort	
	**Post-Matching**			
	**N**	**%**	**N**	**%**
**Total**	1,699,913	100.0%	1,699,913	100.0%
**Sex**				
Female	1,017,413	59.9%	1,017,413	59.9%
**Age at Index**				
Mean (SD)	75.4 (6.8)		75.4 (6.8)	
Median (IQR)	74.0 (70.0—80.0)		74.0 (70.0—80.0)	
Min (Max)	65 (111)		65 (111)	
**Duration of Post-Index Enrollment (months)**				
Mean (SD)	40.6 (17.3)		44.1 (17.0)	
Median (IQR)	40.2 (25.1—57.2)		46. (29.4—59.1)	
Min (Max)	1 (72)		0 (72)	
**Charlson Comorbidity Index**				
Mean (SD)	1.7 (2.5)		0.7 (1.5)	
Median (IQR)	1 (0 – 3)		0 (0 – 1)	
Min (Max)	0 (24)		0 (20)	
**Comorbidities**				
Depression	296,097	17.4%	56,258	3.3%
Osteoporosis	280,462	16.5%	178,721	10.5%
Traumatic brain injury	62,318	3.7%	16,672	1.0%
Alzheimer’s disease and related dementia	144,834	8.5%	31,812	1.9%
Chronic pain	108,468	6.4%	19,222	1.1%
Obstructive sleep apnea	113,161	6.7%	0	0.0%
**Index Treatment**				
Benzodiazepines	494,655	29.1%		
Trazodone	581,117	34.2%		
Zolpidem ER	8,196	0.5%		
Zolpidem IR	615,945	36.2%		

*Abbreviations*: *ER* Extended release, *IQR* Interquartile range, *IR* Immediate release, *Max* Maximum, *Min* Minimum, *SD* Standard deviation

### Fall outcomes

**Fig. 1:**
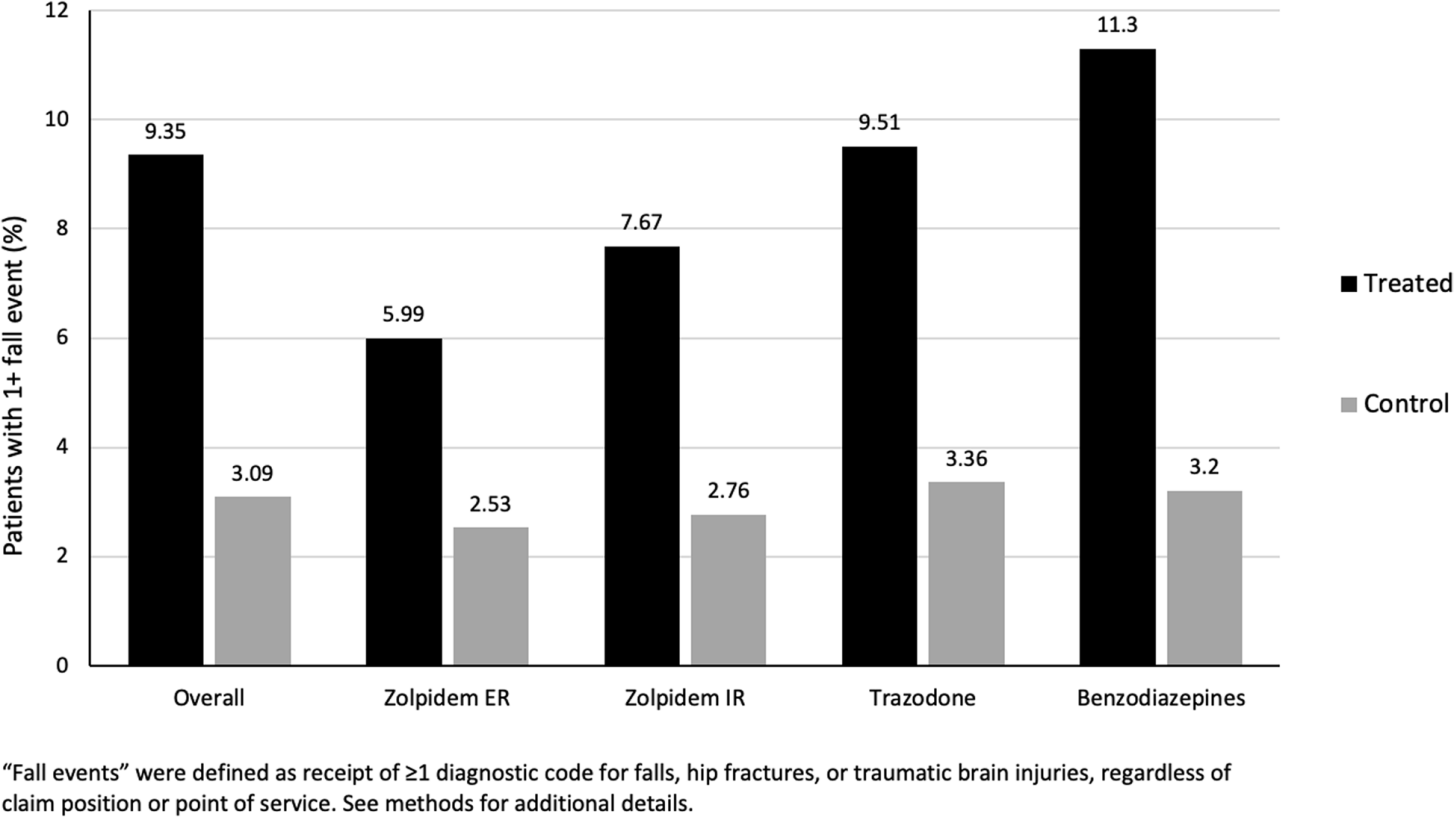
Percentage of patients with 1 + falls in the overall and stratified insomnia treated and matched control cohorts

Relative to non-sleep disordered controls, patients with treated insomnia were more likely to have experienced ≥ 1 fall events in the 12 months post-index (9.4 vs 3.1%; Fig. 1). Among the insomnia treated cohort, a greater proportion of patients receiving benzodiazepines (11.3%) or trazodone (9.5%) experienced a fall event relative to the proportions of patients receiving zolpidem IR (7.7%) or zolpidem ER (6.0%).

Relative to non-sleep disordered controls, beneficiaries with treated insomnia with ≥ 1 falls experienced more falls per enrollee (2.0 [SD 2.2] vs. 1.6 [SD 1.7]). This pattern was consistent across all individual insomnia treatments studied. The mean number of fall events per patient with one or more falls was highest among patients receiving trazodone. The mean time to first fall in patients with one or more fall event was 154.2 days (SD 111.8) in the insomnia treated group (*n* = 160,465) and 176.1 days (SD 107.9) in the matched control group (*n* = 53,011).

Among patients with treated insomnia, the adjusted incident rate (IR) for a fall event was 9.2 per 100 person years (PY; 95% CI 9.1 to 9.3); among controls this IR was 4.1 per 100 PY (95% CI 4.0 to 4.1). Among patients with treated insomnia, the incidence rate ratio (IRR) for a fall event was 2.26 (95% CI 2.2 to 2.3). Relative to the matched control cohort, fall events were more common in each treatment cohort studied. Relative to the adjusted IR for patients receiving zolpidem IR (8.2 per 100 PY) or zolpidem ER (7.2 per 100 PY), the adjusted IR was higher for patients receiving benzodiazepines (10.0 per 100 PY) or trazodone (9.8 per 100 PY).

**Fig. 2:**
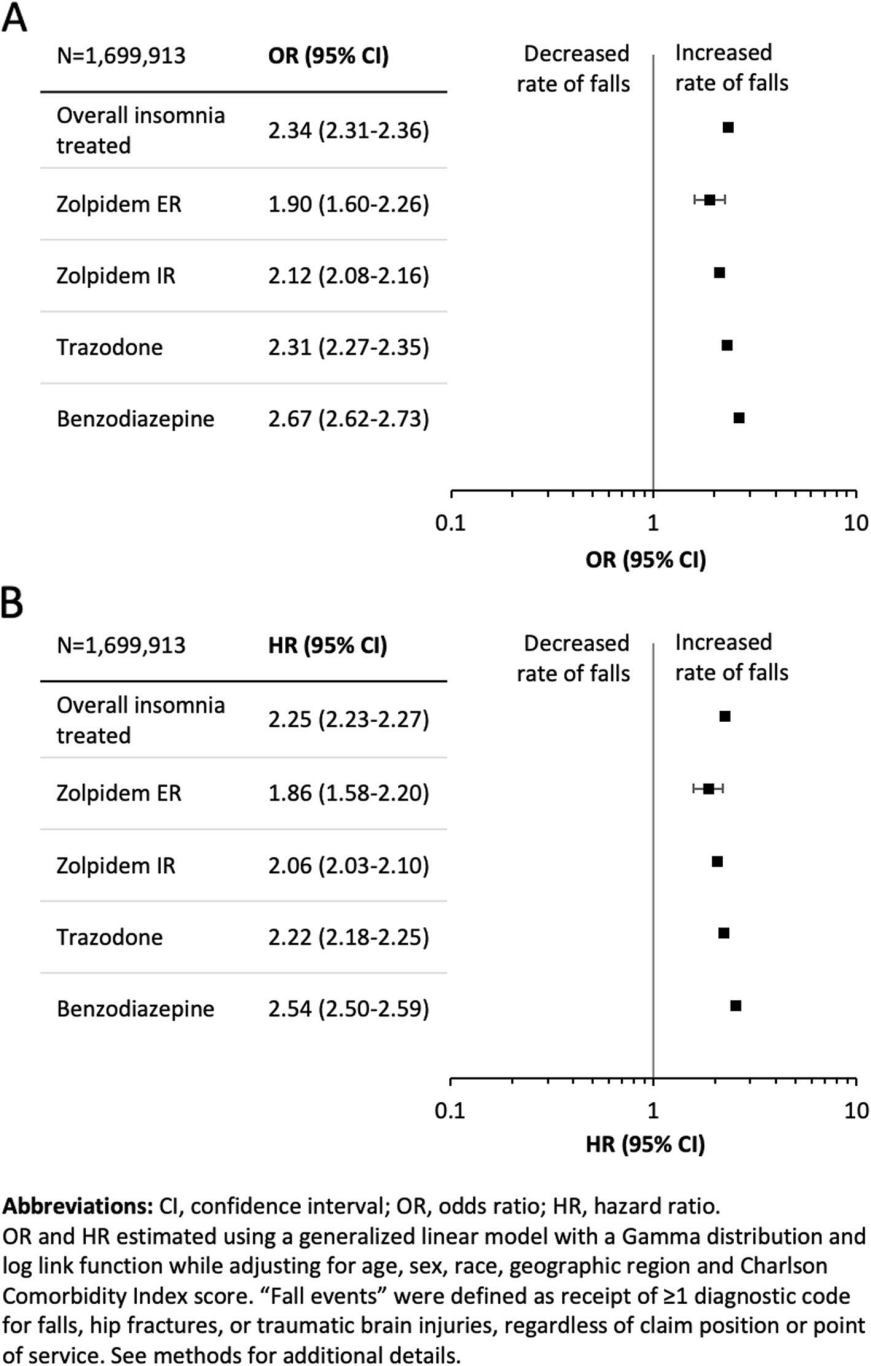
**a** Odds ratio (OR) for fall events in the insomnia treated cohort. **b** Hazard ratio (HR) for fall events in the insomnia treated cohort

Relative to non-sleep disordered controls, the OR for a fall event in the overall insomnia treated cohort was 2.3 (95% CI: 2.1, 2.4)) (Fig. 2A). Relative to the control cohort, the HR for the first fall after the index date for the overall insomnia treated cohort was 2.3 (95% CI: 2.23, 2.3) (Fig. 2B). Each value was highest among patients receiving benzodiazepines followed by trazodone. Note that OR and HR patterns for overall treatment group and specific medications were quite similar.

### Mortality

Relative to non-sleep disordered controls, beneficiaries with treated insomnia were significantly more likely to experience all-cause mortality in the 12 months post-index (0.3% vs 0.02%; *p* < 0.001). Among beneficiaries with treated insomnia who experienced a fall event, relative to non-sleep disordered controls, all-cause mortality was significantly more likely (prevalence 0.9% vs. 0.1%, *p* < 0.001).

Patients receiving zolpidem ER, zolpidem IR, trazodone, or benzodiazepines were significantly more likely to experience all-cause mortality, relative to the respective controls (all *p* < 0.001). Among individuals who experienced falls, relative to non-sleep-disordered controls, patients receiving zolpidem IR, trazodone, or benzodiazepines were significantly more likely to experience all-cause mortality (*p* < 0.001). Further, in this population, all-cause mortality was greater among patients receiving trazodone (0.4%) than among patients receiving benzodiazepines (0.3%), zolpidem IR (0.3%) or zolpidem ER (0.2%).

### All-cause HCRU

Relative to non-sleep disordered controls, patients with treated insomnia demonstrated greater mean inpatient visits per month (0.1 vs 0.1; OR 1.3; 95% CI 1.3 to 1.3; *p* < 0.001), and a longer estimated mean length of inpatient stay (10.8 days vs. 7.1 days; OR 1.5; 95% CI 1.5 to 1.5; *p* < 0.001) (Table 2). Relative to controls, patients with treated insomnia also demonstrated significantly greater mean ED visits (0.2 vs. 0.1; OR 1.3; 95% CI 1.3 to 1.3; *p* < 0.001) and outpatient visits (0.6 vs. 0.4; OR 1.5; 95% CI 1.5 to 1.4; *p* < 0.001) (Table 2).

**Table 2:** All cause healthcare resource utilization in the insomnia treated and matched control cohorts

Category	Overall Patient			
	**N (%)**	**Estimated Mean**	**Estimated Mean** **Ratio with 95% CI**	***P*-value**^a^
**Inpatient Visits**				
** Number of visits per month**				
Treated Patients	315,679 (18.6%)	0.13	1.29 (1.28,1.30)	< .001
Matched Control Cohort	83,826 (4.9%)	0.10		
** Length of inpatient stay among those with inpatient visits (days)**				
Treated Patients	315,679 (18.6%)	10.81	1.52 (1.52, 1.53)	< .001
Matched Control Cohort	83,826 (4.9%)	7.10		
**ED Visits**				
** Number of visits per month**				
Treated Patients	374,441(22.0%)	0.15	1.30 (1.297,1.31)	< .001
Matched Control Cohort	146,947 (8.6%)	0.12		
**Outpatient Visits**				
** Number of visits per month**				
Treated Patients	868,655 (51.1%)	0.56	1.49 (1.48,1.49)	< .001
Matched Control Cohort	595,020 (35.0%)	0.37		

*Abbreviations*: *CI*, Confidence interval, *ED* Emergency department

^a^Mean estimated using a generalized linear model with a Gamma distribution and log link function while adjusting for age, sex, race, geographic region and Charlson Comorbidity Index score

Empty cells represent values not available for analysis. OR adjusted model is not feasible, due to the small sample size

Patients with 1 + MVA, or 1 + fall and MVA, represented a negligible population and were excluded as motor vehicle insurance may have covered healthcare costs

Relative to non-sleep disordered controls, patients with treated insomnia demonstrated greater estimated mean inpatient visits, ED visits, and outpatient visits, which were similar across the stratified insomnia treated groups (Supplementary Table 1). Estimated mean ratios (EMRs) for inpatient visits and ED visits were greatest among patients receiving benzodiazepines, while the EMR for outpatient visits was greatest among patients receiving trazodone (Supplementary Table 1).

### All-cause cost

Relative to matched non-sleep disordered controls, patients with treated insomnia demonstrated greater estimated mean total costs per patient per month (PPPM) ($967.0 vs $454.6; EMR 2.1; 95% CI, 2.1 to 2.1; *p* < 0.001) (Table 3). Patients with treated insomnia also demonstrated greater inpatient costs, ED costs, and outpatient costs (all *p* < 0.001) (Table 3).

**Table 3:** All cause costs in the insomnia treated and matched control cohorts

Category	Overall Patient			
	**N (%)**	**Estimated Mean**	**Estimated Mean Ratio with 95% CI**	***P*-value**^a^
**Total Cost (PPPM)**				
Treated Patients	1,699,913 (100.0%)	$967	2.13 (2.12, 2.13)	< .001
Matched Control Cohort	1,603,929 (94.4%)	$455		
**Medical Cost (PPPM)**				
** Inpatient Costs**				
** All patients**				
Treated Patients	315,679 (18.6%)	$1,912	1.25 (1.24, 1.26)	< .001
Matched Control Cohort	83,826 (4.9%)	$1,529		
** Patients with no fall**^b^ **or MVA events**^c^				
Treated Patients	226,035 (13.3%)	$1,761	1.21 (1.20, 1.22)	< .001
Matched Control Cohort	66,618 (3.9%)	$1,454		
** Patients with 1 + fall**^b^				
Treated Patients	87,298 (5.1%)	$2,343	1.27 (1.25, 1.29)	< .001
Matched Control Cohort	16,602 (1.0%)	$1,848		
** ED Costs**				
** All patients**				
Treated Patients	374,441 (22.0%)	$134	1.39 (1.38, 1.39)	< .001
Matched Control Cohort	146,947 (8.6%)	$97		
** Patients with no fall**^b^ **or MVA events**^c^				
Treated Patients	256,246 (15.1%)	$125	1.32 (1.31, 1.33)	< .001
Matched Control Cohort	111,059 (6.5%)	$94		
** Patients with 1 + fall**^b^				
Treated Patients	113,905 (6.7%)	$158	1.49 (1.47, 1.50)	< .001
Matched Control Cohort	34,016 (2.0%)	$106		
** Outpatient Costs**				
** All patients**				
Treated Patients	868,655 (51.1%)	$292	1.82 (1.81, 1.83)	< .001
Matched Control Cohort	595,020 (35.0%)	$161		
** Patients with no fall**^b^ **or MVA events**^c^				
Treated Patients	727,581 (42.8%)	$279	1.81 (1.80, 1.82)	< .001
Matched Control Cohort	552,413 (32.5%)	$154		
** Patients with 1 + fall**^b^				
Treated Patients	136,150 (8.0%)	$380	1.51 (1.49, 1.54)	< .001
Matched Control Cohort	40,523 (2.4%)	$251		

*Abbreviations*: *CI* Confidence interval, *PPPM* Per patient per month, *MVA* Motor vehicle accident

^a^Healthcare costs estimated using a generalized linear model with a Gamma distribution and log link function while adjusting for age, sex, race, geographic region and Charlson Comorbidity Index score. Empty cells represent values not available for analysis. OR adjusted model is not feasible, due to the small sample size. Patients with 1 + MVA, or 1 + fall and MVA, represented a negligible population and were excluded as motor vehicle insurance may have covered healthcare costs

^b^ “Fall events” were defined as receipt of ≥ 1 diagnostic code for falls, hip fractures, or traumatic brain injuries (TBIs), regardless of claim position or point of service. See methods for additional details

^c^ “MVA events” were defined as receipt of ≥ 1 diagnostic code for a MVA. See methods for additional details

Relative to matched non-sleep disordered controls, the mean total cost PPPM was highest among patients receiving benzodiazepines (EMR 2.4; 95% Cl 2.3, 2.4 *p* < 0.001) (Supplementary Table 2). Relative to matched controls, patients with treated insomnia receiving zolpidem IR, trazodone, or benzodiazepines demonstrated significantly higher inpatient costs PPPM (all *p* < 0.001), but no differences were observed for patients receiving zolpidem ER (Supplementary Table 2). This trend was also observed for patients who experienced a fall event: Patients with treated insomnia demonstrated significantly greater ED costs PPPM and outpatient costs PPPM (all *p* < 0.001). Similar relations were observed among patients experiencing fall events and receiving each index treatment (ED costs and outpatient costs, *p* < 0.01) (Supplementary Table 2).

## Discussion

In this national Medicare administrative claims data study, older adult beneficiaries taking common insomnia medications demonstrated significantly elevated risk of falls, all-cause mortality, HCRU, and costs compared with non-sleep disordered controls. In addition to highlighting adverse outcomes associated with common insomnia treatments, our most novel findings pertain to substantial risks associated with trazodone, which is often perceived as a safe, albeit off-label, approach to insomnia medication treatment [22]. Note, however, as this study was observational and not a randomized controlled trial, the results show association and not causality. Specifically, residual confounding due to potential misclassification of medication exposure and lack of availability of clinical variables such as severity of insomnia and other underlying illnesses cannot be ruled out.

This study’s findings are consistent with two previous studies among older adults which suggested increased risk of falls associated with trazodone use [23] and comparable risk of fall-related injury associated with use of low-dose trazodone or benzodiazepines [24]. Very few studies have examined the impact of trazodone in insomnia, particularly among older adults. Roth and colleagues conducted a small, placebo-controlled study and found trazodone to be associated with impairments in next day balance and arm muscle strength among older adults with insomnia [25]. Low-dose trazodone is commonly prescribed off-label for insomnia, yet it remains understudied and controversial. Indeed, the most common side effects associated with trazodone include drowsiness and dizziness [26]. Nonetheless, many prescribers perceive trazodone as a relatively safe treatment [22] – and some payers may require off-label use of low-dose trazodone as first line therapy for insomnia – although AASM Clinical Guidelines do not recommend trazodone due to limited efficacy/safety study data [10]. AASM asserts that this physician perception of trazodone as a “safer” sleep-promoting agent and consequent prescribing practices of those physicians have engendered the widespread use of trazodone for insomnia [10]. In this vein, findings from the current national study add an important population health perspective to the literature.

Among our 100% sample of Medicare beneficiaries, relative to non-sleep disordered controls, patients with treated insomnia demonstrated a 2.0- to 2.5-fold greater risk of falls. These results are consistent with and build upon previous studies showing a higher risk of falls among older adults treated for insomnia [27–31]. In most analyses, relative to patients receiving zolpidem IR or zolpidem ER, patients receiving benzodiazepines demonstrated the greatest risk of falls, followed by patients receiving trazodone. Increased risk of falls associated with benzodiazepines and zolpidem have been extensively reported in patients with insomnia [32–37]. The risk is particularly salient among older adults [33] for whom sedative hypnotic medications are inappropriate per published guidelines [9]. Consistent with prior reports [16], we found these potentially inappropriate medicines to be prescribed frequently among older adults [38].

Present results suggest that relative to non-sleep disordered controls, patients with treated insomnia were significantly more likely to have experienced all-cause mortality. Likewise, relative to patients who did not experience falls, individuals who experienced falls were observed to be significantly more likely to die from any cause. Importantly, relative to patients receiving other insomnia medications, patients receiving trazodone had a higher rate of death. Although prior research has compared mortality between patients receiving trazodone and antipsychotics [25], to our knowledge, this is the first study demonstrating an increased risk of mortality in patients receiving trazodone for insomnia.

In addition to increased risk of falls and death, relative to non-sleep disordered controls, patients with treated insomnia also demonstrated worse economic outcomes. Patients with treated insomnia demonstrated more than twice the rates of inpatient and ED visits. Similarly, patients with treated insomnia demonstrated more than twice the overall healthcare costs, even after controlling for confounding variables, including geographic region, medical and psychiatric comorbidity burden, age, race and sex. The observations of higher HCRU and costs for patients with insomnia are consistent with previous reports and add to a growing body of literature regarding the economic burden of insomnia and health economic aspects of insomnia treatments. In one large study, ED visits and physician visits were 50% and 120% higher, respectively for patients with insomnia than for those without [39, 40]. In another study of Medicare beneficiaries, ED visits were higher among insomnia treated patients in the year following diagnosis [41]. Inpatient costs, followed by outpatient costs, were the primary drivers of total costs both pre- and post-insomnia diagnosis, in both treated and untreated cohorts. Surprisingly, this previous Medicare analysis showed no significant difference in total costs between the treated and untreated cohorts during the 12 months prior and 12 months post-insomnia diagnosis [42]. In another study, relative to non-sleep disordered controls, patients with untreated insomnia demonstrated $63,607 (95% CI: $60,532, $66,685) higher all-cause healthcare utilization and costs in the 11 months prior to insomnia diagnosis or matched index date, with inpatient care accounting for most of the healthcare use and costs (2019 USD) [43].

Falls are associated with substantial economic burden. Depending on the severity of the fall, costs per fall can range from $1,596 to $10,913, and costs for fall-related hospitalizations can range from $10,052 to $42,840 (2010 USD) [43]. Total annual costs of insomnia in the US are estimated at $77 to $116 billion, with $75 to $100 billion of these costs being indirect [2]. Thus, present results are consistent with prior studies demonstrating a substantial public health burden and elevated direct medical costs associated with both insomnia treatment and falls.

Our study possesses strengths. Most important, we performed a comprehensive, large-scale analysis of the impact of treated insomnia on falls and other adverse outcomes among older adults. Second, in addition to being large, our Medicare sample is highly generalizable, representing 100% of Medicare fee-for-service beneficiaries in the US. Third, we considered the most widely prescribed insomnia treatments, including low-dose trazodone, which is frequently used off-label to treat insomnia. Fourth, we employed multiple measures to maximize the specificity of our operational definitions, such as excluding patients receiving benzodiazepines with a concurrent diagnosis of anxiety.

At the same time, our methodology has limitations for consideration. Most important, we were unable to assess some clinical variables including insomnia symptoms or severity, daytime function, and quality of life. Second, we did not match on specific comorbidities, concomitant medication use or previous fall history, but we did control for comorbidity burden using the Charlson Comorbidity Index in the adjusted generalized linear models. Claims data limitations did not allow us to control for patient frailty or severity of illness. It has been suggested that these patients may use hypnotics more often for reasons such as stress and fear because of an underlying severe illness. Third, we assessed only falls that required medical attention, and we were unable to assess falls that resulted in death. Fourth, there is currently no validated algorithm to identify patients with insomnia in administrative claims data, and insomnia is underreported and underdiagnosed. Thus, we relied on prescription drug claims for on- or off-label insomnia treatments to identify the patients with treated insomnia. Dosing was limited for trazodone to ≤ 100 mg without requiring an accompanying insomnia diagnosis to further ensure its use for insomnia in the absence of a diagnosis code. Claims data do not contain information on whether or when the patient took their prescription medication, only the fill date. Fifth, we did not control for extent of exposure (dosage and persistence) in the analysis; effects were attributed to first index medication with ≥ 5 days’ supply. Medication exposure at the time of fall was not ascertained. Sixth, we were unable to assess over-the-counter (OTC) insomnia treatments, certain prescription insomnia medications, or non-pharmacological treatments (ie, cognitive-behavioral treatment). Given the nature of our data source (and our inability to assess use of OTC medication), we were unable to control for untreated insomnia, which has itself been associated with increased risk of falls and HCRU [30, 43]. Finally, administrative claims methodology design precludes determination of causality. Lastly, insomnia patients could differ from the control group beyond exposure to the insomnia drugs of interest, namely with respect to variables that we could not control (eg, disease severity, health status).

In summary, this national analysis of 100% Medicare claims examined outcomes for older adult patients treated with commonly prescribed medications for insomnia (ie, benzodiazepines, trazodone, zolpidem IR, zolpidem ER). Compared with non-sleep disordered controls, treated adult patients demonstrated significantly increased risk of falls, all-cause mortality, and adverse economic outcomes. Although prescribers may perceive trazodone as a lower-risk alternative for treatment of insomnia, this study’s findings suggest that trazodone, like benzodiazepines and zolpidem, was associated with higher risk of falls and higher healthcare resource utilization compared to controls. Further, risk varied by insomnia medication, with benzodiazepines and trazodone typically being associated with the worst outcomes. Note, however, that this was an observational study rather than a randomized controlled trial so the results of this study are associative rather than causal. Future research should seek to identify patients at greatest risk of adverse outcomes from commonly prescribed insomnia treatments and to match these individuals with safe, effective alternatives. Most important, these results highlight the need for insomnia treatments which are suitable for use in older adults with insomnia and are not associated with an increased risk of falls.

## Supplementary Information

**Additional file 1**: **Supplementary Figure 1a and b**. Attrition. **Supplementary Table 1a and 1b**. HCRU by treatment. **Supplementary Table 2a and 2b**. Costs by treatment.

## Data Availability

The data used in this study are available from the Centers for Medicare and Medicaid Services (CMS) data warehouse, but restrictions apply to the availability of these data, which were used under license for the current study, and so are not publicly available. Data are, however, available from the corresponding author upon reasonable request and with permission of CMS.

## References

[1] Bergen G, Stevens MR, Burns ER (2016). Falls and fall injuries among adults aged >/=65 Years - United States, 2014. MMWR Morb Mortal Wkly Rep.

[2] Heinrich S, Rapp K, Rissmann U, Becker C, König HH (2010). Cost of falls in old age: a systematic review. Osteoporos Int.

[3] Croke L (2020). Beers criteria for inappropriate medication use in older patients: an update from the AGS. Am Fam Physician.

[4] American Psychiatric Association (2013). Diagnostic and Statistical Manual of Mental Disorders.

[5] Roy AN, Smith M (2010). Prevalence and cost of insomnia in a state Medicaid fee-for-service population based on diagnostic codes and prescription utilization. Sleep Med.

[6] Bertisch SM, Herzig SJ, Winkelman JW, Buettner C (2014). National use of prescription medications for insomnia: NHANES 1999–2010. Sleep.

[7] Atkin T, Comai S, Gobbi G (2018). Drugs for insomnia beyond benzodiazepines: pharmacology, clinical applications, and discovery. Pharmacol Rev.

[8] Mokhar A, Tillenburg N, Dirmaier J, Kuhn S, Härter M, Verthein U (2018). Potentially inappropriate use of benzodiazepines and z-drugs in the older population—analysis of associations between long-term use and patient-related factors. PeerJ.

[9] American Geriatrics Society (2019). American geriatrics society 2019 updated AGS beers criteria® for potentially inappropriate medication use in older adults. J Am Geriatr Soc.

[10] Sateia MJ, Buysse DJ, Krystal AD, Neubauer DN, Heald JL (2017). clinical practice guideline for the pharmacologic treatment of chronic insomnia in adults: an American Academy of sleep medicine clinical practice guideline. J Clin Sleep Med : JCSM.

[11] Newton R (1981). The side effect profile of trazodone in comparison to an active control and placebo. J Clin Psychopharmacol.

[12] Jaffer KY, Chang T, Vanle B (2017). Trazodone for insomnia: a systematic review. Innov Clin Neurosci.

[13] Wong J, Murray Horwitz M, Bertisch SM, Herzig SJ, Buysse DJ, Toh S (2020). Trends in dispensing of zolpidem and low-dose trazodone among commercially insured adults in the United States, 2011–2018. JAMA.

[14] Maust DT, Blow FC, Wiechers IR, Kales HC, Marcus SC (2017). National trends in antidepressant, benzodiazepine, and other sedative-hypnotic treatment of older adults in psychiatric and primary care. J Clin Psychiatry.

[15] Ford ES, Wheaton AG, Cunningham TJ, Giles WH, Chapman DP, Croft JB (2014). Trends in outpatient visits for insomnia, sleep apnea, and prescriptions for sleep medications among US adults: findings from the national ambulatory medical care survey 1999–2010. Sleep.

[16] Albrecht JS, Wickwire EM, Vadlamani A, Scharf SM, Tom SE (2019). Trends in insomnia diagnosis and treatment among Medicare beneficiaries, 2006–2013. Am J Geriatr Psychiatry.

[17] Aschkenasy MT, Rothenhaus TC (2006). Trauma and falls in the elderly. Emerg Med Clin North Am.

[18] Binder S (2002). Injuries among older adults: the challenge of optimizing safety and minimizing unintended consequences. Inj Prev.

[19] SGIM. Medicare Claims Data. Available at: https://www.sgim.org/communities/research/dataset-compendium/medicare-claims-data.

[20] U.S. Bureau of Labor Statistics. Measuring Price Change in the CPI: Medical care. Available at: https://www.bls.gov/cpi/factsheets/medical-care.htm. Accessed August 26th.

[21] Charlson ME, Pompei P, Ales KL, MacKenzie CR (1987). A new method of classifying prognostic comorbidity in longitudinal studies: development and validation. J Chronic Dis.

[22] Schroeck JL, Ford J, Conway EL (2016). Review of safety and efficacy of sleep medicines in older adults. Clin Ther.

[23] Macri JC, Iaboni A, Kirkham JG (2017). Association between antidepressants and fall-related injuries among long-term care residents. Am J Geriatr Psychiatry.

[24] Bronskill SE, Campitelli MA, Iaboni A (2018). Low-dose trazodone, benzodiazepines, and fall-related injuries in nursing homes: a matched-cohort study. J Am Geriatr Soc.

[25] Roth AJ, McCall WV, Liguori A (2011). Cognitive, psychomotor and polysomnographic effects of trazodone in primary insomniacs. J Sleep Res.

[26] Bayer AJ, Pathy MS, Ankier SI (1983). Pharmacokinetic and pharmacodynamic characteristics of trazodone in the elderly. Br J Clin Pharmacol.

[27] Cauley JA, Hovey KM, Stone KL (2019). Characteristics of Self-Reported Sleep and the Risk of Falls and Fractures: The Women's Health Initiative (WHI). J Bone Miner Res.

[28] Chen TY, Lee S, Buxton OM. A Greater Extent of Insomnia Symptoms and Physician-Recommended Sleep Medication Use Predict Fall Risk in Community-Dwelling Older Adults. Sleep. 2017;40(11). 10.1093/sleep/zsx142 29029240

[29] Zhang Y, Cifuentes M, Gao X, Amaral G, Tucker KL (2017). Age- and gender-specific associations between insomnia and falls in Boston Puerto Rican adults. Qual Life Res.

[30] Avidan AY, Fries BE, James ML, Szafara KL, Wright GT, Chervin RD (2005). Insomnia and hypnotic use, recorded in the minimum data set, as predictors of falls and hip fractures in Michigan nursing homes. J Am Geriatr Soc.

[31] Stone KL, Blackwell TL, Ancoli-Israel S (2014). Sleep disturbances and risk of falls in older community-dwelling men: the outcomes of sleep disorders in older men (MrOS Sleep) study. J Am Geriatr Soc.

[32] Avidan AY, Palmer LA, Doan JF, Baran RW (2010). Insomnia medication use and the probability of an accidental event in an older adult population. Drug Healthc Patient Saf.

[33] Glass J, Lanctôt KL, Herrmann N, Sproule BA, Busto UE (2005). Sedative hypnotics in older people with insomnia: meta-analysis of risks and benefits. BMJ (Clinical research ed).

[34] Kolla BP, Lovely JK, Mansukhani MP, Morgenthaler TI (2013). Zolpidem is independently associated with increased risk of inpatient falls. J Hosp Med.

[35] Seppala LJ, Wermelink A, de Vries M (2018). Fall-risk-increasing drugs: a systematic review and meta-analysis: II. psychotropics. J Am Med Dir Assoc.

[36] Tom SE, Wickwire EM, Park Y, Albrecht JS (2016). Nonbenzodiazepine sedative hypnotics and risk of fall-related injury. Sleep.

[37] Machado FV, Louzada LL, Cross NE, Camargos EF, Dang-Vu TT, Nóbrega OT (2020). More than a quarter century of the most prescribed sleeping pill: systematic review of zolpidem use by older adults. Exp Gerontol.

[38] Slaney H, MacAulay S, Irvine-Meek J, Murray J (2015). Application of the beers criteria to alternate level of care patients in hospital inpatient units. Can J Hosp Pharm.

[39] Watt JA, Gomes T, Bronskill SE (2018). Comparative risk of harm associated with trazodone or atypical antipsychotic use in older adults with dementia: a retrospective cohort study. CMAJ.

[40] Ruxton K, Woodman RJ, Mangoni AA (2015). Drugs with anticholinergic effects and cognitive impairment, falls and all-cause mortality in older adults: a systematic review and meta-analysis. Br J Clin Pharmacol.

[41] DiBonaventura M, Richard L, Kumar M, Forsythe A, Flores NM, Moline M (2015). The association between insomnia and insomnia treatment side effects on health status, work productivity, and healthcare resource use. PLoS One.

[42] Wickwire EM, Vadlamani A, Tom SE, Johnson AM, Scharf SM, Albrecht JS (2020). Economic aspects of insomnia medication treatment among Medicare beneficiaries. Sleep.

[43] Wickwire EM, Tom SE, Scharf SM, Vadlamani A, Bulatao IG, Albrecht JS (2019). Untreated insomnia increases all-cause health care utilization and costs among Medicare beneficiaries. Sleep.

