# Supplementary material for: Falls, healthcare resources and costs in older adults with insomnia treated with zolpidem, trazodone, or benzodiazepines
Source: BMC Geriatr. 2022 Jun 4;22:484. doi: 10.1186/s12877-022-03165-6 (PMC9166444; doi:10.1186/s12877-022-03165-6)
Supplement: Supplementary file 1 — Additional file 1: Supplementary Figure 1a and b. Attrition. Supplementary Table 1a and 1b. HCRU by treatment. Supplementary Table 2a and 2b. Costs by treatment. [file 12877_2022_3165_MOESM1_ESM.docx]

# Supplementary File 1

Supplementary Figure 1a and b: Attrition

Supplementary Table 1a and 1b: HCRU by treatment

Supplementary Table 2a and 2b: Costs by treatment

**Supplementary Figure 1a - Cohort attrition: insomnia treated cohort**

At least 1 medication of interest with an FDA-approved indication for insomnia treatment OR with Trazodone treatment (strength less or equal to 100 MG) OR beneficiaries with at least 1 other off-label insomnia treatment coupled with at least 1 insomnia diagnosis code within the 12 months prior to treatment date (including the treatment date) in Medicare claims from 1 Jan 2012 to 31 Dec 2016.

**(n = 6,794,952)**

With Medicare claims for a single insomnia treatment with > 5 days supply

(Index Date: the earliest fill date).

**(n = 6,682,179)**

Age ≥ 65 years at Index Date.

**(n = 6,634,604)**

At least 12 months of continuous Medicare enrollment with full medical and drug coverage before the Index Date.

**(n = 5,033,794)**

Continuous Medicare enrollment with full medical and drug coverage for at least 12 months post-index date, or until death, whichever occurs earlier.

**(n = 4,494,248)**

Without presence of a Medicare claim for a fill of the any treatment(s) within the 12-month pre-Index period.

**(n = 2,806,955)**

Exclude beneficiaries with an index prescription for benzodiazepine and an anxiety diagnosis in the 12 months baseline.

**(n = 2,758,367)**

Trazodone

**(n = 922,118)**

Benzodiazepines

**(n = 820,813)**

Stratified by index treatment.

Zolpidem ER

**(n = 13,272)**

Zolpidem IR

**(n = 1,002,164)**

**Supplementary Figure 1b - Cohort attrition: matched control**

No evidence of insomnia treatment OR insomnia diagnosis OR sleep-related disorders in Medicare claims from 1 Jan 2011 to 31 Dec 2017.

**(n = 3,250,015)**

Matching to Treated Insomnia Cohort based on age and sex (the Index Date for members of the matched control cohort will be the same as their matched insomnia cases).

**(n = 2,257,245)**

Beneficiaries age ≥ 65 years at Index Date.

**(n = 2,246,336)**

Beneficiaries with at least 12 months of continuous Medicare enrollment with full medical and drug coverage before the Index Date.

**(n = 1,723,095)**

Beneficiaries are required to have continuous Medicare enrollment with full medical and drug coverage for at least 12 months post-index date, or until death, whichever occurs earlier.

(**n = 1,699,913)**

Trazodone

**(n = 581,117)**

Benzodiazepines

**(n = 494,665)**

Stratified by index treatment.

Zolpidem ER

**(n = 8,196)**

Zolpidem IR

**(n = 615,945)**

**Supplemental Table 1a: Adjusted Healthcare Resource Utilization for Treated Insomnia Cohort and Matched Control Cohort within 12 Months Post-Index**

|  | **Zolpidem ER** | | | | **Zolpidem IR** | | | | |
| --- | --- | --- | --- | --- | --- | --- | --- | --- | --- |
| **Category** | **N (%)** | **Estimated Mean** | **Estimated Mean Ratio with 95%CI** | **P-value^1^** | **N (%)** | **Estimated Mean** | | **Estimated Mean Ratio with 95%CI** | **P-value^1^** |
| **Inpatient Visits** |  |  |  |  |  |  | |  |  |
| **Number of visits per month** |  |  |  |  |  |  | |  |  |
| Treated Patients | 1,110 (13.5%) | 0.13 | 1.16 (1.05,1.29) | 0.01 | 108,911 (17.68%) | 0.13 | | 1.28 (1.27,1.30) | <.001 |
| Matched Control Cohort | 372 (4.5%) | 0.11 |  |  | 29,373 (4.77%) | 0.10 | |  |  |
| **Length of inpatient stay among those with inpatient visits (days)** | | | | |  | |  | | |
| Treated Patients | 1,110 (13.5%) | 11.59 | 1.31 (1.26,1.37) | <.001 | 108,911 (17.68%) | 10.27 | | 1.46 (1.45,1.47) | <.001 |
| Matched Control Cohort | 372 (4.5%) | 8.84 |  |  | 29,373 (4.77%) | 7.04 | |  |  |
| **ED Visits** |  |  |  |  |  |  | |  |  |
| **Number of visits per month** |  |  |  |  |  |  | |  |  |
| Treated Patients | 1,388 (16.9%) | 0.15 | 1.21 (1.11,1.32) | <.001 | 123,687 (20.08%) | 0.15 | | 1.25 (1.24,1.26) | <.001 |
| Matched Control Cohort | 627 (7.7%) | 0.12 |  |  | 51,180 (8.31%) | 0.12 | |  |  |
| **Outpatient Visits** |  |  |  |  |  |  | |  |  |
| **Number of visits per month** |  |  |  |  |  |  | |  |  |
| Treated Patients | 3,962 (48.3%) | 0.54 | 1.43 (1.40,1.46) | <.001 | 308,230 (50.04%) | 0.54 | | 1.46 (1.46,1.47) | <.001 |
| Matched Control Cohort | 2,785 (34.0%) | 0.38 |  |  | 213,511 (34.66%) | 0.37 | |  |  |
| **Abbreviations:** CI, confidence interval; ED, emergency department.  ^1^P-value to compare treated and matched control cohort's estimated means were obtained from generalized linear model (GLM) with Poisson distribution and log link while adjusting for age, sex, race, geographic region and Charlson Comorbidity Index score. | | | | | | | | | |

**Supplemental Table 1b: Adjusted Healthcare Resource Utilization for Treated Insomnia Cohort and Matched Control Cohort within 12 Months Post-Index**

|  | **Trazodone** | | | | | **Benzodiazepines** | | | | | |
| --- | --- | --- | --- | --- | --- | --- | --- | --- | --- | --- | --- |
| **Category** | **N (%)** | **Estimated Mean** | **Estimated Mean Ratio with 95%CI** | | **P-value^1^** | **N (%)** | | **Estimated Mean** | **Estimated Mean Ratio with 95%CI** | | **P-value^1^** |
| **Inpatient Visits** |  |  |  | |  |  | |  |  | |  |
| **Number of visits per month** |  |  |  | |  |  | |  |  | |  |
| Treated Patients | 98,067 (16.88%) | 0.13 | 1.29 (1.27,1.30) | | <.001 | 107,591 (21.75%) | | 0.13 | 1.30 (1.29,1.32) | | <.001 |
| Matched Control Cohort | 29,308 (5.04%) | 0.10 |  | |  | 24,773 (5.01%) | | 0.10 |  | |  |
| **Length of inpatient stay among those with inpatient visits (days)** | | | |  | | |  | | |  | |
| Treated Patients | 98,067 (16.88%) | 10.94 | 1.54 (1.53, 1.55) | | <.001 | 107,591 (21.75%) | | 11.30 | 1.57 (1.56, 1.58) | | <.001 |
| Matched Control Cohort | 29,308 (5.04%) | 7.11 |  | |  | 24,773 (5.01%) | | 7.20 |  | |  |
| **ED Visits** |  |  |  | |  |  | |  |  | |  |
| **Number of visits per month** |  |  |  | |  |  | |  |  | |  |
| Treated Patients | 118,638 (20.42%) | 0.15 | 1.32 (1.31,1.33) | | <.001 | 130,728 (26.43%) | | 0.16 | 1.35 (1.33,1.36) | | <.001 |
| Matched Control Cohort | 51,937 (8.94%) | 0.12 |  | |  | 43,203 (8.73%) | | 0.12 |  | |  |
| **Outpatient Visits** |  |  |  | |  |  | |  |  | |  |
| **Number of visits per month** |  |  |  | |  |  | |  |  | |  |
| Treated Patients | 267,303 (46.00%) | 0.59 | 1.52 (1.52,1.52) | | <.001 | 289,160 (58.46%) | | 0.55 | 1.48 (1.48,1.48) | | <.001 |
| Matched Control Cohort | 205,152 (35.30%) | 0.39 |  | |  | 173,572 (35.09%) | | 0.37 |  | |  |
| **Abbreviations**: CI, confidence interval; ED, emergency department.  ^1^P-value to compare treated and matched control cohort's estimated means were obtained from generalized linear model (GLM) with Poisson distribution and log link while adjusting for age, sex, race, geographic region and Charlson Comorbidity Index score. | | | | | | | | | | | |

**Supp. Table 2a: Adjusted^1^ PPPM Healthcare Costs for Treated Insomnia Cohort and Matched Control Cohort within 12 Months Post-Index**

|  | **Zolpidem ER** | | | | **Zolpidem IR** | | | |
| --- | --- | --- | --- | --- | --- | --- | --- | --- |
| **Category** | **N (%)** | **Estimated Mean** | **Estimated Mean Ratio with 95% CI** | **P-value^1^** | **N (%)** | **Estimated Mean** | **Estimated Mean Ratio with 95% CI** | **P-value^1^** |
| **Total Cost (PPPM)** |  |  |  |  |  |  |  |  |
| Treated Patients | 8,196 (100.0%) | 1,099 | 2.37 (2.27, 2.47) | <.001 | 615,945 (100.00%) | 979 | 2.15 (2.14, 2.16) | <.001 |
| Matched Control Cohort | 7,702 (94.0%) | 464 |  |  | 579,865 (94.14%) | 456 |  |  |
| **Medical Cost (PPPM)** |  |  |  |  |  |  |  |  |
| **Inpatient Costs** |  |  |  |  |  |  |  |  |
| **All patients** |  |  |  |  |  |  |  |  |
| Treated Patients | 1,110 (13.5%) | 2,465 | 1.17 (1.01, 1.35) | 0.04 | 108,911 (17.68%) | 1,945 | 1.28 (1.26, 1.30) | <.001 |
| Matched Control Cohort | 372 (4.5%) | 2,116 |  |  | 29,373 (4.77%) | 1,526 |  |  |
| **Patients with no fall or MVA events** |  |  |  |  |  |  |  |  |
| Treated Patients | 880 (10.7%) | 2,059 | 1.18 (1.01, 1.38) | 0.04 | 82,301 (13.36%) | 1,808 | 1.24 (1.22, 1.26) | <.001 |
| Matched Control Cohort | 305 (3.7%) | 1,743 |  |  | 23,861 (3.87%) | 1,457 |  |  |
| **Patients with 1+ fall** |  |  |  |  |  |  |  |  |
| Treated Patients | 220 (2.7%) | 3,838 | 1.14 (0.78, 1.68) | 0.50 | 25,779 (4.19%) | 2,395 | 1.33 (1.28, 1.37) | <.001 |
| Matched Control Cohort | 66 (0.8%) | 3,362 |  |  | 5,282 (0.86%) | 1,806 |  |  |
| **ED Costs** |  |  |  |  |  |  |  |  |
| **All patients** |  |  |  |  |  |  |  |  |
| Treated Patients | 1,388 (16.9%) | 127 | 1.40 (1.27, 1.53) | <.001 | 123,687 (20.08%) | 130 | 1.37 (1.35, 1.38) | <.001 |
| Matched Control Cohort | 627 (7.7%) | 91 |  |  | 51,180 (8.31%) | 95 |  |  |
| **Patients with no fall or MVA events** |  |  |  |  |  |  |  |  |
| Treated Patients | 1,038 (12.7%) | 124 | 1.40 (1.26, 1.55) | <.001 | 88,879 (14.43%) | 122 | 1.31 (1.30, 1.33) | <.001 |
| Matched Control Cohort | 501 (6.1%) | 89 |  |  | 39,422 (6.40%) | 93 |  |  |
| **Patients with 1+ fall** |  |  |  |  |  |  |  |  |
| Treated Patients | 328 (4.0%) | 136 | 1.30 (1.06, 1.59) | 0.01 | 33,267 (5.40%) | 154 | 1.49 (1.45, 1.52) | <.0001 |
| Matched Control Cohort | 119 (1.5%) | 105 |  |  | 11,022 (1.79%) | 104 |  |  |
| **Outpatient Costs** |  |  |  |  |  |  |  |  |
| **All patients** |  |  |  |  |  |  |  |  |
| Treated Patients | 3,962 (48.3%) | 296 | 2.02 (1.88, 2.17) | <.001 | 308,230 (50.04%) | 311 | 1.94 (1.92, 1.95) | <.001 |
| Matched Control Cohort | 2,785 (34.0%) | 147 |  |  | 213,511 (34.66%) | 161 |  |  |
| **Patients with no fall or MVA events** |  |  |  |  |  |  |  |  |
| Treated Patients | 3,515 (42.9%) | 279 | 2.05 (1.91, 2.21) | <.001 | 266,129 (43.21%) | 299 | 1.94 (1.92, 1.95) | <.001 |
| Matched Control Cohort | 2,623 (32.0%) | 136 |  |  | 199,571 (32.40%) | 154 |  |  |
| **Patients with 1+ fall** |  |  |  |  |  |  |  |  |
| Treated Patients | 423 (5.2%) | 377 | 1.41 (1.08, 1.84) | 0.01 | 40,309 (6.54%) | 403 | 1.62 (1.58, 1.67) | <.001 |
| Matched Control Cohort | 157 (1.9%) | 268 |  |  | 13,118 (2.13%) | 248 |  |  |
| **Abbreviations:** CI, confidence interval; ED, emergency department; MVA, motor vehicle accident; PPPM, per patient per month.  ^1^ Healthcare costs were estimated using a generalized linear model with a Gamma distribution and log link function while adjusting for age, sex, race, geographic region and Charlson Comorbidity Index score. Patients with $0 or negative cost were assigned $0.001 cost.  Patients with 1+ MVA, or 1+ fall and MVA, represented a negligible population and were excluded as motor vehicle insurance may have covered costs. | | | | | | | | |

**Supp Table 2b: Adjusted^1^ PPPM Healthcare Costs for Treated Insomnia Cohort and Matched Control Cohort within 12 Months Post-Index**

|  | **Trazodone** | | | | **Benzodiazepines** | | | |
| --- | --- | --- | --- | --- | --- | --- | --- | --- |
| **Category** | **N (%)** | **Estimated Mean** | **Estimated Mean Ratio with 95% CI** | **P-value^1^** | **N (%)** | **Estimated Mean** | **Estimated Mean Ratio with 95% CI** | **P-value^1^** |
| **Total Cost (PPPM)** |  |  |  |  |  |  |  |  |
| Treated Patients | 581,117 (100.00%) | 897 | 1.93 (1.92, 1.94) | <.001 | 494,655 (100.00%) | 1,035 | 2.35 (2.34, 2.36) | <.001 |
| Matched Control Cohort | 549,324(94.53%) | 464 |  |  | 467,038 (94.42%) | 441 |  |  |
| **Medical Cost (PPPM)** |  |  |  |  |  |  |  |  |
| **Inpatient Costs** |  |  |  |  |  |  |  |  |
| **All patients** |  |  |  |  |  |  |  |  |
| Treated Patients | 98,067 (16.88%) | 1,832 | 1.21 (1.19, 1.23) | <.001 | 107,591 (21.75%) | 1,936 | 1.25 (1.23, 1.27) | <.001 |
| Matched Control Cohort | 29,308 (5.04%) | 1,511 |  |  | 24,773 (5.01%) | 1,547 |  |  |
| **Patients with no fall or MVA events** |  |  |  |  |  |  |  |  |
| Treated Patients | 67,429 (11.60%) | 1,675 | 1.17 (1.15, 1.19) | <.001 | 75,425 (15.25%) | 1,771 | 1.21 (1.18, 1.23) | <.001 |
| Matched Control Cohort | 22,873 (3.94%) | 1,430 |  |  | 19,579 (3.96%) | 1,469 |  |  |
| **Patients with 1+ fall** |  |  |  |  |  |  |  |  |
| Treated Patients | 29,961 (5.16%) | 2,221 | 1.21 (1.18, 1.25) | <.001 | 31,338 (6.34%) | 2,389 | 1.27 (1.23, 1.31) | <.001 |
| Matched Control Cohort | 6,225 (1.07%) | 1,832 |  |  | 5,029 (1.02%) | 1,888 |  |  |
| **ED Costs** |  |  |  |  |  |  |  |  |
| **All patients** |  |  |  |  |  |  |  |  |
| Treated Patients | 118,638 (20.42%) | 138 | 1.39 (1.38, 1.41) | <.001 | 130,728 (26.43%) | 135 | 1.40 (1.38, 1.41) | <.001 |
| Matched Control Cohort | 51,937 (8.94%) | 99 |  |  | 43,203 (8.73%) | 96 |  |  |
| **Patients with no fall or MVA events** |  |  |  |  |  |  |  |  |
| Treated Patients | 78,259 (13.47%) | 127 | 1.33 (1.31, 1.35) | <.001 | 88,070 (17.80%) | 125 | 1.32 (1.31, 1.34) | <.001 |
| Matched Control Cohort | 38,704 (6.66%) | 96 |  |  | 32,432 (6.56%) | 95 |  |  |
| **Patients with 1+ fall** |  |  |  |  |  |  |  |  |
| Treated Patients | 39,185 (6.74%) | 162 | 1.46 (1.43, 1.48) | <.001 | 41,125 (8.31%) | 158 | 1.51 (1.48, 1.54) | <.001 |
| Matched Control Cohort | 12,640 (2.18%) | 112 |  |  | 10,235 (2.07%) | 104 |  |  |
| **Outpatient Costs** |  |  |  |  |  |  |  |  |
| **All patients** |  |  |  |  |  |  |  |  |
| Treated Patients | 267,303 (46.00%) | 272 | 1.69 (1.67, 1.70) | <.001 | 289,160 (58.46%) | 292 | 1.81 (1.80, 1.83) | <.001 |
| Matched Control Cohort | 205,152 (35.30%) | 161 |  |  | 173,572 (35.09%) | 161 |  |  |
| **Patients with no fall or MVA events** |  |  |  |  |  |  |  |  |
| Treated Patients | 218,558 (37.61%) | 259 | 1.67 (1.66, 1.69) | <.001 | 239,379 (48.39%) | 277 | 1.80 (1.79, 1.82) | <.001 |
| Matched Control Cohort | 189,474 (32.61%) | 155 |  |  | 160,745 (32.50%) | 153 |  |  |
| **Patients with 1+ fall** |  |  |  |  |  |  |  |  |
| Treated Patients | 47,327 (8.14%) | 346 | 1.43 (1.39, 1.47) | <.001 | 48,091 (9.72%) | 386 | 1.51 (1.47, 1.55) | <.001 |
| Matched Control Cohort | 15,010 (2.58%) | 242 |  |  | 12,238 (2.47%) | 256 |  |  |
| **Abbreviations:** CI, confidence interval; ED, emergency department; MVA, motor vehicle accident; PPPM, per patient per month.  ^1^ Healthcare costs were estimated using a generalized linear model with a Gamma distribution and log link function while adjusting for age, sex, race, geographic region and Charlson Comorbidity Index score. Patients with $0 or negative cost were assigned $0.001 cost.  Patients with 1+ MVA, or 1+ fall and MVA, represented a negligible population and were excluded as motor vehicle insurance may have covered costs. | | | | | | | | |
| **Abbreviations:** CI, confidence interval; ED, emergency department; MVA, motor vehicle accident; PPPM, per patient per month.  ^1^ Healthcare costs will be estimated using a generalized linear model with a Gamma distribution and log link function. Assign $0.001 to patients with $0 or negative cost.  Empty cells represent values not available for analysis. Or adjusted model is not feasible, due to the small sample size.  Patients with 1+ MVA, or 1+ fall and MVA, represented a negligible population and were excluded as motor vehicle insurance may have covered costs. | | | | | | | | |
